# Nutritional and Lifestyle Psychiatry: The Role of Nutrition and Lifestyle in the Onset, Prevention and Management of Mental Disorders

**DOI:** 10.3390/nu17162575

**Published:** 2025-08-08

**Authors:** Antonios Dakanalis, Constantinos Giaginis

**Affiliations:** 1 Department of Mental Health, Fondazione IRCSS San Gerardo dei Tintori, Via G.B. Pergolesi 33, 20900 Monza, Italy; antonios.dakanalis@unimib.it; 2 Department of Medicine and Surgery, University of Milano Bicocca, Via Cadore 38, 20900 Monza, Italy; 3 Department of Food Science and Nutrition, School of Environment, University of Aegean, 81100 Myrina, Greece

In the last few years, nutritional and lifestyle psychiatry has emerged as a novel scientific topic, highlighting the impact of nutrition and lifestyle in the onset, prevention and management of diverse mental diseases [1,2]. A gradually increasing number of studies are evaluating whether diet and nutrition could affect the risk of mental disorders through neuroinflammation, the microbiome and the impact of specific dietary factors and nutrients [3,4]. Diverse types of diet (e.g., intermittent fasting, ketogenic diet, Mediterranean diet, veganism, etc.) and specific micronutrients, herbs and supplements have been examined for their potential beneficial effects on human mental health [3,4]. Several recent studies also assess the potential impact and efficacy of healthy lifestyle practices, from exercise and meditation to sleep and stress management, in preventing, reducing and managing mental health symptoms, as well as in helping psychiatric patients to address weight gain issues [5,6].

In view of the above considerations, a pilot cross-sectional study determined the effect of overweight and obesity on the psychosocial functioning and physical activity levels of children and adolescents. This study found that overweight and obesity in children and adolescents were associated with a negative body image, low self-esteem, unhealthy eating habits and low levels of physical activity [7]. Another cross-sectional study was designed to evaluate the psychological health status of children aged 3–7 years and analyze the correlation between dietary behaviors, lifestyle and psychological problems. This study highlighted the urgent necessity for targeted interventions addressing insufficient dietary diversity, distracted eating, excessive screen time and unhealthy sleep habits to improve the psychological well-being of preschool children [8]. A longitudinal study explored the potential associations between healthy eating habits, resilience, insomnia, and internet addiction through a cross-lagged panel analysis of Chinese college students. This study provided higher-level evidence and important implications for interventions focused on reducing internet addiction among college students, developing healthy eating habits and improving resilience and sleep health [9].

A randomized, double-blind, placebo-controlled pilot study was designed to evaluate the clinical effects of monotherapy based on omega-3 polyunsaturated fatty acids (n-3 PUFAs) in patients with major depressive disorder. This study supported evidence that n-3 PUFA-based monotherapy may improve depression and potentially serve as an alternative option for MDD patients [10]. Another study explored the potential impact of total added sugar and sugar-sweetened beverage consumption on depression, as well as their potential interactions with chronic stress. This study showed that both total sugar consumption and sugar-sweetened beverage consumption at baseline predicted depressive symptoms one month later. Nevertheless, only sugar-sweetened beverage intake was a significant predictor of depression after controlling for stress, possibly because stress is related to diet quality [11]. Additionally, a recent study explored whether socioeconomic and health variables may be associated with dietary assessment in a population with high rates of social deprivation and whether a relationship may exist between dietary assessment and depressive symptoms. This study showed that sociodemographic variables can affect nutritional habits. In fact, women who lived in rural areas and were limited to a vocational education had significantly poorer diets. Moreover, men, younger men, smokers, and those without chronic diseases were characterized by a poorer dietary assessment. Additionally, women who exhibited a better dietary assessment were significantly more likely to have lower scores on the questionnaire assessing the occurrence of depression symptoms [12].

Notably, a nationwide study in Korea investigated the association between dietary riboflavin and suicidal ideation. This study supported evidence for a non-linear inverse relationship between riboflavin intake and suicidal ideation, with notable variations by sex and age. These findings suggested that modifying dietary riboflavin intake may be a crucial strategy for reducing suicide risk [13]. Another study underscored the essential role of self-efficacy as the most influential factor affecting health-promoting behaviors in individuals with schizophrenia. This study suggested that enhancing self-efficacy may emerge as a crucial element in the design and implementation of intervention programs aimed at improving health-promoting behaviors in individuals with schizophrenia [14]. Moreover, a cross-sectional study evaluated the effect of dietary patterns and physical activity on the well-being of patients with diabetes mellitus. This study showed that regular exercise and the consumption of fruits, dairy products, and white meat may exert beneficial effects on the mental well-being of patients with diabetes mellitus [15].

Conclusively, ongoing research supports significant evidence that modifying nutritional behavior and lifestyle factors may act as preventing agents against mental health disorders. However, there is a strong demand to perform longitudinal studies, as well as interventional surveys, to confirm the impact of nutrition and lifestyle in the onset, prevention and management of mental disorders.
